# Cost of TB services: approach and summary findings of a multi-country study (Value TB)

**DOI:** 10.5588/ijtld.22.0096

**Published:** 2022-11-01

**Authors:** S. Sweeney, Y. V. Laurence, L. Cunnama, G. B. Gomez, I. Garcia-Baena, P. Bhide, T. J. Capeding, S. Chatterjee, I. Chikovani, H. Eyob, A. Kairu, M. M. Terefe, N. Shengelia, M. Toshniwal, N. Saadi, E. Bergren, A. Vassall

**Affiliations:** 1 Global Health Economics Centre, London School of Hygiene & Tropical Medicine, London, UK; 2 University of Cape Town, Cape Town, South Africa; 3 Global Access, International Aids Vaccine Initiative, Amsterdam, The Netherlands; 4 World Health Organization, Geneva, Switzerland; 5 The George Institute for Global Health, New Delhi, India; 6 University of the Philippines Manila, Manila, The Philippines; 7 Curatio International Foundation, Tbilisi, Georgia; 8 Armauer Hansen Research Institute, Addis Ababa, Ethiopia; 9 Health Economics Research Unit, Kenya Medical Research Institute–Wellcome Trust Research Programme, Nairobi, Kenya; 10 University of Oslo, Oslo, Norway

**Keywords:** unit cost, methods, costing

## Abstract

**BACKGROUND:**

There are currently large gaps in unit cost data for TB, and substantial variation in the quality and methods of unit cost estimates. Uncertainties remain about sample size, range and comprehensiveness of cost data collection for different purposes. We present the methods and results of a project implemented in Kenya, Ethiopia, India, The Philippines and Georgia to estimate unit costs of TB services, focusing on findings most relevant to these remaining methodological challenges.

**METHODS:**

We estimated financial and economic unit costs, in close collaboration with national TB programmes. Gold standard methods included both top-down and bottom-up approaches to resource use measurement. Costs are presented in 2018 USD and local currency unit.

**RESULTS:**

Cost drivers of outputs varied by service and across countries, as did levels of capacity inefficiency. There was substantial variation in unit cost estimates for some interventions and high overhead costs were observed. Estimates were subject to sampling uncertainty, and some data gaps remain.

**CONCLUSION:**

This paper describes detailed methods for the largest TB costing effort to date, to inform prioritisation and planning for TB services. This study provides a strong baseline and some cost estimates may be extrapolated from this data; however, regular further studies of similar quality are needed to add estimates for remaining gaps, or to add new or changing services and interventions. Further research is needed on the best approach to extrapolation of cost data. Costing studies are best implemented as partnerships with policy makers to generate a community of mutual learning and capacity development.

## Abstract (fr)

**CONTEXTE:**

Il existe actuellement d’importantes lacunes en matière de données relatives aux coûts unitaires de la TB, et la qualité ainsi que les méthodes utilisées pour estimer les coûts unitaires varient considérablement. Des incertitudes persistent quant à la taille d’échantillon, la gamme et l’exhaustivité des recueils de données de coûts nécessaires à différentes fins. Nous présentons les méthodes et les résultats d’un projet mis en oeuvre au Kenya, en Éthiopie, en Inde, aux Philippines et en Géorgie visant à estimer les coûts unitaires des services antituberculeux, en se concentrant sur les résultats les plus utiles aux défis méthodologiques qui subsistent.

**MÉTHODES:**

Nous avons estimé les coûts unitaires financiers et économiques, en collaboration étroite avec les programmes nationaux de lutte contre la TB. Les méthodes de référence comprenaient à la fois des méthodes descendantes et ascendantes de mesure de l’utilisation des ressources. Les coûts sont présentés en dollars américains 2018 (USD) ainsi que dans la devise locale.

**RÉSULTATS:**

Les facteurs de coût des résultats varient selon les services et les pays, tout comme les niveaux d’inefficacité des capacités. Les estimations des coûts unitaires de certaines interventions variaient considérablement et des frais indirects élevés ont été observés. Les estimations étaient sujettes à l’incertitude de l’échantillonnage, et certaines lacunes subsistent en matière de données.

**CONCLUSION:**

Cet article décrit les méthodes détaillées du plus grand effort d’évaluation des coûts de la TB jamais réalisé à ce jour, afin de façonner la priorisation et la planification des services antituberculeux. Cette étude fournit un point de référence solide, à partir duquel certaines estimations des coûts peuvent être extrapolées. D’autres études de même qualité devront toutefois être réalisées afin de fournir les estimations relatives aux lacunes restantes, ou d’ajouter de nouveaux services/interventions ou des services/interventions en proie à des changements. D’autres études sont aussi nécessaires afin de déterminer la meilleure approche pour extrapoler les données de coûts. Il convient de réaliser les études de coûts dans le cadre de partenariats avec les décideurs politiques, afin de créer une communauté évoluant dans un cycle d’apprentissage mutuel et de développement des capacités.

Ending TB will require substantial investment for many years to come. Current resourcing for TB is highly constrained, requiring difficult decisions to be made globally and within national TB programmes (NTPs). One of the main challenges to this endeavour is the dearth of data on the costs of TB services and interventions, which is needed to estimate the resource requirements to achieve TB control and evaluate investment priorities.^1^–^6^ Recent systematic reviews have highlighted a gap in data on the unit costs of treatment of drug-susceptible TB (DS-TB) and TB that is multidrug-resistant or rifampicinresistant (MDR/RR-TB).^2^,^3^,^7^,^8^ Where unit cost data does exist, it is often out of date, does not utilise a representative sample of facilities and often does not reflect the ‘real world’ costs of implementation at scale. Cost data gaps are also wide for TB diagnostics and drug susceptibility testing.^5^,^6^,^9^ Finally, there is almost no recent cost data available for ‘new’ approaches to TB control such as enhanced or active case-finding, the treatment of latent TB and improvement of social protection.^10^

A challenge for analysts producing and using cost data is substantial variation in the quality and methods of unit cost estimates.^7^ Although there are textbooks and guideline documents on costing approaches, several key challenges and gaps in costing methods remain. First, given the high costs and logistical challenges of data collection, cost estimation frequently employs convenience sampling methods, and sample sizes are often small. A related challenge is uncertainty about the necessary range and comprehensiveness of cost data collection for different purposes. Pragmatic costing approaches which reduce the level of detail required for certain inputs would reduce the cost of data collection; however, these approaches are not validated. Next, many analysts use a ‘mixed-methods’ approach to estimating resource use, combining ‘top-down’ and ‘bottom-up’ allocation methods for different shared resources, although the degree to which this may influence cost estimates is unclear. Finally, the format of data for sharing varies across studies, often requiring extensive additional analysis when used as secondary data.

The aim of this paper is to present the methods and results of a five-country project (Value TB) funded by the Bill and Melinda Gates Foundation to estimate the unit costs of TB services, focusing on findings most relevant to these remaining methodological challenges. We describe the methods and selected findings for Value TB studies conducted between 2016 and 2018 in India, Kenya, The Philippines, Georgia and Ethiopia.

## Methods and common approach

The overarching aim of Value TB was to enable NTPs and their funders to allocate resources in an efficient and fair way. To achieve this aim, researchers collected data on the average cost per output, or unit cost, of main TB services in each country. Primary cost data were collected on all inputs, including those shared with other programmes or services. Cost estimates were produced for different scales and types of health care providers in each country, as these are known to be key drivers of cost variation.^11^ Data were made available in full online as multi-purpose datasets that could be used for in-country estimates, as a starting point for other countries, and to validate other non- or partially data driven cost estimates.^12^

Value TB also aimed to develop a sustainable framework for TB service cost data collection in each country. Value TB was implemented in each country as a partnership between researchers and the NTPs, with technical support provided by the London School of Hygiene & Tropical Medicine (LSHTM; London, UK), University of Cape Town (UCT; Cape Town, South Africa) and the WHO where needed. National stakeholder groups were established in each country consisting of users and producers of TB cost data, to define priority costing needs and methods gaps, and review results. Cost data collection tools and guidelines were also made available online with the aim for these to be sustainable resources for future data collection efforts.^13^ Following the period of cost data collection, teams reflected on lessons learnt for future TB cost estimation efforts.

## Study logistics

We aimed to use methods that were as ‘gold standard’ as possible within our budget and timeframe. The approximate in-country budget size for country data collection and preliminary analysis was USD3,440 per health facility and USD74,000 per country, excluding capacity building costs of supporting institutions such as LSHTM, UCT and the WHO, which in total for all countries amounted to USD678,103. In all countries, this was the first time such a comprehensive costing study had been carried out by the study teams.

The period of data collection ranged from 6–12 months, with 1–10 months spent on study design and obtaining ethical approval, 3–14 months on data collection and 7–18 months on data cleaning and descriptive analysis. Time frames varied widely across countries according to different ethics processes and local approvals needed, staffing of researchers, availability of health facilities for visitation and complexity of data. Table 1 shows the total time taken for each stage of the project in different settings.

**Table 1:** Value TB sampling and data collection

	The Philippines	Kenya	India	Ethiopia	Georgia
Total new and relapse TB cases notified /100,000 population, 2019 (95% CI)^29^	554 (311–866)	267 (163–396)	193 (132–266)	140 (98–188)	74 (62–87)
Total population, 2019, thousands	108,116	52,573	1,366,417	112,078	3,720
GDP per capita, 2019, USD	3,485.1	1,816.5	2,104.1	857.5	4,769.2
National TB budget, 2019, USD millions^29^	205	81	583	94	13
Total facilities sampled					
Geography: rural	10	9	6	5	5
Geography: urban	18	11	14	21	14
Geography: semi-urban	0	0	0	0	9
Ownership: private	11	2	7	6	15
Ownership: public	17	13	13	19	13
Ownership: NGO/faith-based	0	5	0	1	0
Observations for direct and ancillary services (number of facilities where data collection took place), *n*					
Community services and visits	1 (1)	12 (10)	6 (5)	14 (14)	2 (2)
Inpatient visits	9 (6)	14 (12)	12 (10)	6 (4)	6 (3)
Laboratory tests	105 (25)	117 (20)	114 (20)	249 (26)	135 (21)
Outpatient visits	123 (27)	111 (19)	74 (18)	148 (25)	94 (26)
Other services and visits*	19 (12)	66 (19)	8 (8)	18 (18)	26 (19)
Radiology	18 (15)	20 (11)	14 (13)	17 (12)	25 (19)
Observations for different types of interventions (number of facilities where data collection took place), *n*					
DS-TB treatment	50 (20)	157 (19)	44 (11)	163 (23)	39 (19)
MDR/RR-TB treatment	18 (9)	5 (5)		12 (3)	35 (19)
Active case-finding	1 (1)	20 (15)		11 (10)	26 (21)
BCG vaccination	20 (20)	17 (17)	10 (10)	23 (22)	
Intensified case-finding: cough triage	24 (11)	3 (2)		86 (22)	
Intensified case-finding: screening	52 (20)	70 (18)		82 (21)	
Passive case-finding	73 (27)	80 (19)	38 (18)	101 (25)	26 (19)
TB prevention	7 (7)	37 (19)	5 (5)	31 (22)	2 (2)
Approximate time taken for project stages, months					
Protocol development, ethics applications, and local approvals	2	5	12	5	1
Period of data collection	6	6	10	9	6
Data cleaning	8	12	8	8	4
Descriptive analysis	12	12	5	12	12

* ‘Other’ services and visits included contact tracing, lost to follow-up tracing, provision of vouchers or food baskets and phone consultations.

CI = confidence interval; GDP = gross domestic product; USD = United States dollar; NGO = non-governmental organisation; DS-TB = drug-susceptible TB; MDR/RR-TB = multidrug-resistant/rifampicin-resistant TB; BCG = bacille Calmette-Guérin.

## Sampling methods

Our country-level sampling frame included the 48 countries classified as high-burden countries by the WHO in 2017,^14^ excluding countries with recent cost data for at least 80% of intervention categories, and countries with majority-inpatient treatment. Countries were stratified by income levels, and region, and two countries per strata were identified and approached to assess feasibility and gauge interest in participation. If any country declined to participate, we randomly selected a replacement from other countries in the same strata. Our final country selection includes Kenya, Ethiopia, India, The Philippines and Georgia—representing 36% of the global notified TB burden in 2017.^15^

## Within-country sampling

Our within-country sampling unit was the healthcare facility. Sample sizes were agreed with the funder as the maximum number of facilities feasible within the given budget. Guided by NTPs, we first sampled geographical area for pragmatic reasons. Sampling frames were created from a list of public and private healthcare facilities within the selected geographical areas. A multi-stage stratified random sampling approach included random selection of districts within regions (first stage), followed by a random selection of facilities across strata of facility ownership, scale and urbanicity (second stage). Where facilities provided certain services to a group of smaller centres (e.g., laboratory services), we first sampled ‘‘parent’’ facilities and then ‘‘child’’ facilities. Within-country sampling methods are described further in each country-specific paper in this series.^16^–^20^

## Defining interventions and units

We followed the Global Health Cost Consortium (GHCC) definitions of unit costs for several interventions, including intensified case finding (ICF), active case-finding (ACF), passive case detection, TB treatment, TB prevention and bacille Calmette-Guérin (BCG) vaccination.^21^ Implementation of each intervention varied according to national policy and practice; interventions were defined at each country level, in coordination with the NTP and stakeholders. Unit costs were broken down by target group, platform, phase and regimen type.

## Data collection

Cost data were collected using two standardised tools in MS Excel (MicroSoft, Redmond, WA, USA), developed as part of the Value TB Costing Tool Suite, including a ‘data collection tool’ and a standardised ‘data entry tool’.^13^ We estimated full, real-world financial and economic costs from a health provider perspective, so that costs could be used for financial planning, as well as economic evaluation and priority setting. Cost data collection was retrospective, over a 1-year period to minimise the risk of bias due to seasonal fluctuations in service volume, road conditions or stock-outs.

Resource use was measured using both top-down and bottom-up methods, to allow for comparison and estimation of efficiency. Bottom-up methods capture production process inefficiency and may better characterise variation in practice, while top-down methods may better capture capacity inefficiency, for example, due to down time and wastage.^13^,^22^ We used multiple approaches to estimate resource use, according to costing approach and data availability. For bottom-up estimation of staff time, equipment and supply use, we used observation to estimate levels of input usage for a service where possible. Interviews were used where observation was not feasible within the timeframe for data collection or due to patient confidentiality concerns. For top-down estimates of staff time, we used retrospective data collected through staff timesheets and interviews to allocate the total quantity of inputs used in the facility across services; assumptions were used where timesheets and interviews were not possible.

Local price data sources were used to value traded (e.g., equipment) and non-traded goods (e.g., staff time) in the first instance, using national prices where local prices were unavailable. The local market price was used to reflect the value of donated resources, and the national average wage used to value volunteer time. Capital costs were annuitised over their expected useful life using a standard 3% discount rate.^22^–^24^ The useful life of capital goods was sourced locally in the first instance, and standard values were used where local estimates were unavailable.^13^

All costs are presented in local currency units (LCU), and in United States dollars (USD). We do not report costs in international dollars using purchasing power parity adjustments as currency exchange rates are more representative of prices paid, in line with our aim of facilitating NTP financial planning. We used the average exchange rate for the year of cost data collection to convert costs into USD. Where possible, we used the current value of all resources; in some cases, past expenditures were inflated using the local consumer price index of the country before converting to USD;^25^ this is detailed in country-specific papers in this series.^16^–^20^

## Cost data cleaning and analysis

Data cleaning followed a standard cross-country process (Supplementary Figure S1). The first step required detailed manual review by the study coordinator and investigators for each facility using MS Excel. A data analysis workshop was conducted in January 2019 in Geneva, Switzerland, to allow for cross-country learning. This was followed by pooled analysis using Stata v15 (StataCorp, College Station, TX, USA) to generate per-intervention and per-technology unit cost estimates and identify outlier values requiring further investigation by the in-country team. Data were pooled across countries using Stata v15, and standardised Do-files were supplied to country teams for generation of standard reporting results tables which were included in manuscript appendices.^12^ Descriptive analysis for each country-specific paper was performed by the country team in Stata v15 and MS Excel.

**Figure 1:**
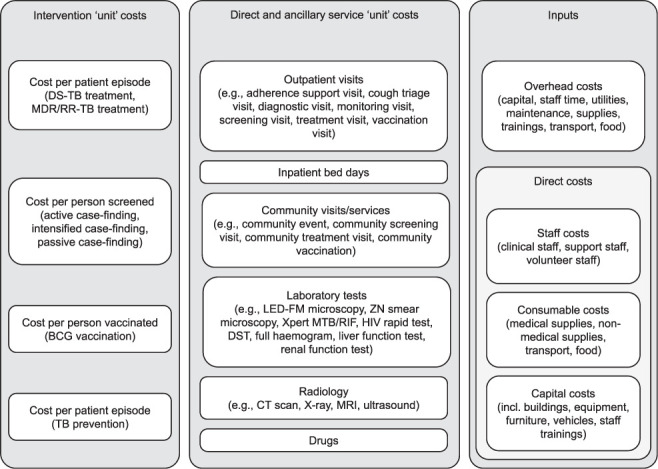
Value TB cost structure. DS-TB = drug-susceptible TB; MDR-TB = multidrug-resistant TB; RR-TB = rifampicin-resistant TB; BCG = bacille Calmette-Guérin; LED-FM = light-emitting diode fluorescence microscopy; ZN = Ziehl-Neelsen; DST = drug susceptibility testing; CT = computerised tomography; MRI = magnetic resonance imaging.

To facilitate use of TB service cost data for multiple purposes, we generated two datasets for public data sharing, using the broad structure illustrated in the GHCC reference case (Figure 1). The first dataset provides estimates of the unit costs of a range of direct and ancillary services (defined per visit or per test), provided by 121 health facilities across five countries.^12^ Unit costs are reported by standard inputs, including staff time, building space, capital, equipment, consumables and overheads.

A second dataset details the unit cost of TB interventions. Unit costs per intervention are estimated as the product of unit costs per direct and ancillary service and the average quantity of each service delivered per episode, defined as relevant for each intervention, e.g., per patient episode of treatment, or per diagnosis. Unit costs per intervention also include the costs of drugs. Traded and non-traded goods were defined in both datasets to facilitate potential extrapolation across settings. All unit cost estimates in the present manuscript were inflated to a base year of 2018.

## SELECTED FINDINGS

### Range and comprehensiveness of cost estimates

Across the five countries, unit cost estimates were generated for 82 different types of direct and ancillary services delivered across 122 facilities. These services were delivered as part of 35 different types of interventions. Table 1 shows the number of observations for each type of service and each type of intervention, and the number of facilities where data collection for each type of service took place.

We were unable to include the estimation of above-facility costs, such as policy, planning and coordination (i.e., TB programme management and supervision), within the Value TB study budget envelope. We were also unable to capture any cost estimates for certain services due to capacity constraints; for example, although surgical interventions are frequently provided as part of TB treatment in Georgia, we did not have team capacity to cost these very complex interventions. In some countries, we were also unable to estimate full unit costs of certain services per patient episode. For example, MDR/RR-TB treatment in India involved multiple referrals to higher-level facilities; therefore, although we were able to estimate the unit cost of most direct and ancillary services involved in providing second-line care, we could not capture the total cost per patient episode of care. Finally, some services were not provided by sampled facilities; for example, active case-finding was rare in Philippines, and intensified case-finding was not conducted by the sampled facilities in Georgia or India. Across countries, few facilities routinely provided community-level and inpatient services.

### Sampling and accuracy of cost estimates

Supplementary Tables S1 and S2 show the mean and coefficient of variation for unit cost estimates per output, by country and method of resource measurement. Although the ‘true’ mean unit cost for TB services in our sample countries is unknown, the coefficient of variation can be used as a measure of precision of estimates; observations with a coefficient of variation greater than 20% of the estimated mean are conventionally considered subject to sampling uncertainty.^26^ Our unit cost estimates had a coefficient of variation over 20% for many services, suggesting that use of these estimates in modelling or other purposes should consider this lack of precision. Coefficients of variation were generally smaller for outpatient services, which could more frequently be directly observed by data collectors, and larger for services that posed logistical challenges to direct observation by researchers, such as certain laboratory services (culture, drug-susceptibility testing), radiology services, community-level services and ‘other’ services, as defined above. Coefficients of variation were also larger for top-down estimates than for bottom-up estimates, reflecting varying levels of capacity efficiency between facilities and suggesting a larger sample is needed for top-down cost estimation.

### Estimation of resource use

Table 2 shows methods for staff time estimation across output types. Across countries, an average of 26% of bottom-up staff time estimates were captured using observation, with the remainders estimated using interviews. Observation was logistically difficult for community-level, inpatient and ‘other’ services; interviews were frequently used to capture resource use for these services. Top-down cost estimates were based on a mix of interviews and timesheets; across countries an average of 37% of top-down staff time estimates were captured through interviews, 48% using time sheets and 15% using assumptions.

**Table 2:** Methods for resource use estimation

	Top-down cost estimates			Bottom-up cost estimates	
	
	Proportion of observations estimated through interview %	Proportion of observations estimated through timesheets %	Proportion of observations estimated through assumption %	Proportion of observations estimated through observation %	Proportion of observations estimated through interview %
Community visits and services	33	36	31	0	100
Inpatient visits	53	24	23	4	91
Laboratory tests				33	67
Outpatient visits	36	49	15	22	78
Other services	35	57	8	5	94
Radiology				43	57

**Figure 2:**
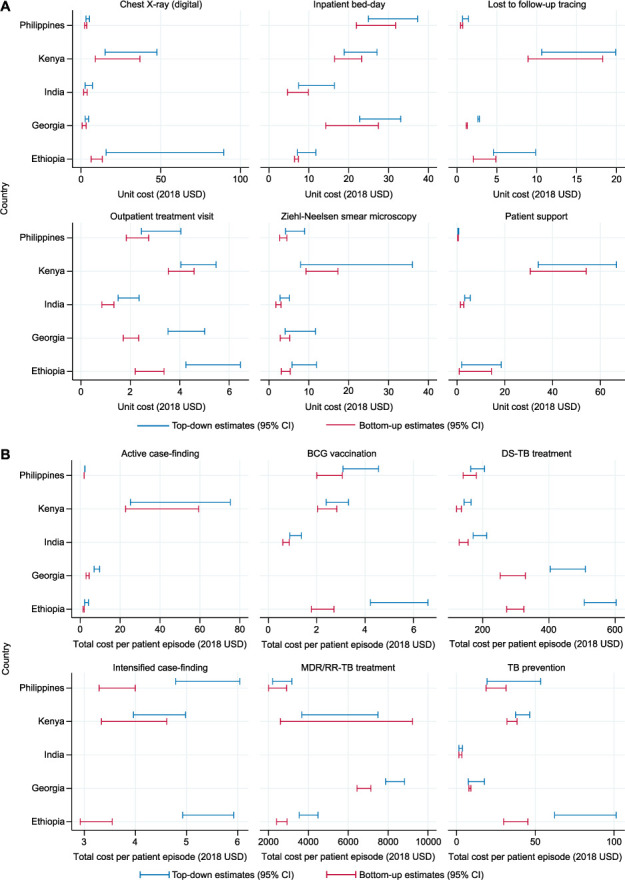
Cost variation by top-down vs. bottom-up estimation methods, by output and intervention type. USD dollar; DS-TB = United States = drug-susceptible TB; MDR-TB = multidrug-resistant TB; RR-TB = rifampicin-resistant TB; CI = confidence interval.

Unit costs estimated using a top-down approach were generally higher than those estimated using a bottom-up approach, reflecting some capacity inefficiency at facilities in all countries. Figure 2 shows the 95% confidence intervals by country for economic unit costs of selected direct and ancillary services, and selected interventions using top-down and bottom-up approaches. The degree of capacity inefficiency varied by output and by facility level and ownership. This was most often attributed to consumable wastage, equipment down time or staff down time in less busy facilities. There were a small number of observations where top-down cost estimates were lower than bottom-up costs—this was usually because researchers were better able to capture some resource use through bottom-up cost estimation approaches, where these resources were not recorded in data sources used for top-down cost estimation. For example, close observation may identify resources that are not part of protocols or laboratory standard operating procedures and may be recorded in budgets but not identified as relevant resources by facility managers.

### Cost drivers and extrapolation

**Figure 3:**
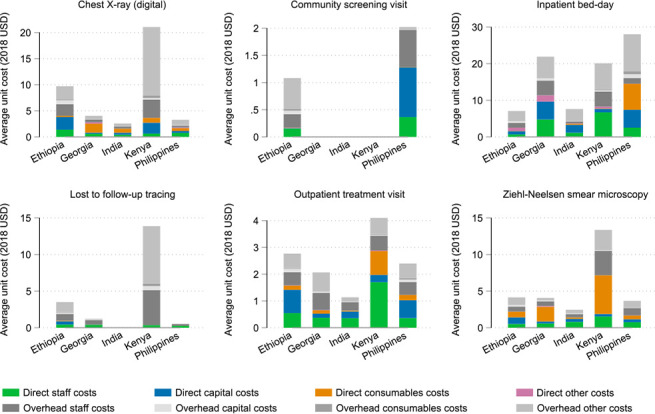
Unit cost of selected direct and ancillary services, by country and input type (bottom-up). USD = United States dollar.

Figure 3 shows the average unit cost for six selected direct and ancillary services by input. Cost drivers of outputs varied by service and across countries. For example, the main driver of direct costs for Ziehl-Neelsen smear microscopy tests was consumables in Kenya, Georgia and The Philippines, staff time in India and capital in Ethiopia. This was due to a combination of differences in operationalisation, prices and laboratory capacity in each different setting. Overhead costs, including administrative staff time, utilities and transport, were high in several settings. Our results suggest that commonly occurring efforts to avoid detailed collection of overheads by assuming a standard inflation of direct costs may vastly underestimate the cost of delivering TB services. This was amplified in some cases by a lack of economies of scale where very few services were delivered in the year of cost estimation, for example, with loss to follow-up tracing in Kenya.

**Figure 4:**
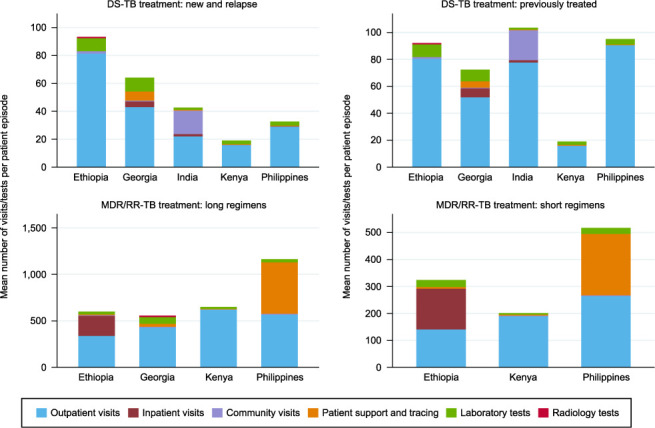
Cross-country variation in treatment practice (2018). Treatment practice data collected in 2018. DS-TB = drug-susceptible TB; MDR/RR-TB = multidrug-resistant/rifampicin-resistant TB.

Unit costs per standard TB intervention also varied according to differences in local prices and models of TB care across countries. Figure 4 shows the average quantity of visits and laboratory/diagnostic tests per patient episode for first- and second-line TB treatment by direct and ancillary service component. India provided more community-level services for DS-TB than other countries, while the Philippines provided more patient support and tracing for MDR/RR-TB patients. The number of visits made to a provider during TB treatment in Kenya was much lower than in other countries. Drug costs also varied across and within countries, with the largest differences seen for MDR/RR-TB treatment; this was partly explained by different sourcing for drug procurement.

## LESSONS LEARNT AND RECOMMENDATIONS

The Value TB project was the largest TB costing effort to date, producing detailed unit cost estimates of a wide range of TB services and interventions. In the five study countries, researchers generated high-quality unit cost estimates with direct application to policy and planning decisions in close collaboration with NTPs. Anonymised per-facility datasets are publicly available online, and include detailed estimates of unit costs and resource use across five countries with different income levels. The datasets provide the range of unit costs and provide new evidence on the gap between near-guideline costs (bottom-up) and real-world expenditure derived costs (top-down) for some services.^12^ Cross-country results also highlight the importance of cost categories such as overhead costs for TB services, which are often overlooked. The data collection tools and guidance are also available online.^13^ These resources together provide a good foundational framework for TB cost data collection and use of unit cost data to inform prioritisation and planning for TB services.

We publish here the costs of conducting our study and the length of time it took. Large scale ‘gold standard’ costing studies are expensive, and most of the cost is incurred by capacity building activities. In total as part of this project, 35 researchers and NTP personnel were trained in methods for costing and cost analysis. Given the high cost and lengthy time investment, it may not be feasible for all high TB burden countries to carry out such intense costing methods on a regular basis. More rapid estimation methods may be good interim measures where lengthy costing studies cannot be implemented; our datasets provide a good baseline to inform these interim estimates. They can be used 1) to identify those inputs that are most important to cost; 2) to validate simpler methods; 3) to make efficiency adjustments to normative costing approaches. They can also be updated to reflect price and quantity changes across settings and time. This dataset should therefore be seen as a global public good that can be used to validate rapid country-based costing efforts.

However, there remain some large gaps in unit costs and methods which should be addressed going forward. First, although this costing exercise was well-funded and supported, we were still unable to estimate certain costs due to budget and time constraints, including above-facility costs, surgeries or adverse events. Although we included cost of inpatient care, our sampling strategy excluded countries where hospitalisation for TB treatment was lengthy so we did not estimate costs of long-term hospitalisation. In India, multiple referrals to higher-level facilities meant we were unable to capture the cost per patient episode of MDR/RR-TB treatment; a study aiming to estimate this accurately would require the resources and ethical approval to track patients to their final treating facility. TB technologies are also evolving, and we were unable to capture all areas of evolving programmatic concern, such as active case detection, which still varies substantially in process and implementation across programmes and settings.^10^ Although Value TB has provided a strong baseline, we would recommend regular further studies of similar quality to add unit cost estimates for these remaining gaps, or to add new or changing services and interventions taking into consideration TB policy cycles.

Second, there is a need for improved guidance on how to use these studies to estimate costs for different purposes. Our data suggest that some selected local data may be needed to ensure appropriate extrapolation, rather than simply applying unit costs, as we observed substantial within- and across-country variation in unit cost estimates and high overhead costs for some services. Although there were differences in key cost drivers across countries, the major differences between countries were driven by staff and overhead costs, and consumables for laboratory services; these would therefore be areas of key focus for rapid cost estimation.

The study has highlighted comparative usefulness of top-down vs. bottom-up cost estimates to inform different policy questions. In Kenya, bottom-up costing approaches helped policymakers identify bottlenecks in active case-finding implementation. In Georgia, top-down cost estimates were used to inform tariff settings. In most countries, comparing top-down and bottom-up estimates also helped with estimates of capacity efficiency, informing policy decisions for better allocation of resources. Differences between top-down and bottom-up estimates are discussed in further detail in other papers in this series.^16^–^20^

Finally, we generated substantial capacity for future cost data collection and unit cost estimation, with a community of researchers with TB cost data collection skills.^27^ The detailed effort made by Value TB investigators to collect unit cost data was essential to interpreting the results and informing policy decisions; however, there was a learning curve with Value TB tools due to the detailed approach needed to capture complex data. Value TB was best implemented as a process of mutual learning between in-country researchers, across country teams, and with regular input from supporting partners. In-country partners led the engagement with the eventual user of the cost data (i.e., NTP) and ensured the data met the NTP’s needs. Going forward, it is important to continue to develop capacity and community of mutual support.
