# Supplementary material for: Cost of TB services: approach and summary findings of a multi-country study (Value TB)
Source: Int J Tuberc Lung Dis. 2022 Nov 1;26(11):1006–15. doi: 10.5588/ijtld.22.0096 (PMC9621303; doi:10.5588/ijtld.22.0096)
Supplement: Supplementary file 1 [file iutld_ijtld_21.0096_supplementarydata1.pdf]

http://dx.doi.org/10.5588/ijtld.21.0096

SUPPLEMENTARY DATA

Costs of TB services: approach and summary findings of a multi-country study (Value TB)

Supplementary Figure S1 Flow diagram illustrating data cleaning and pooling process in the Value TB costing study

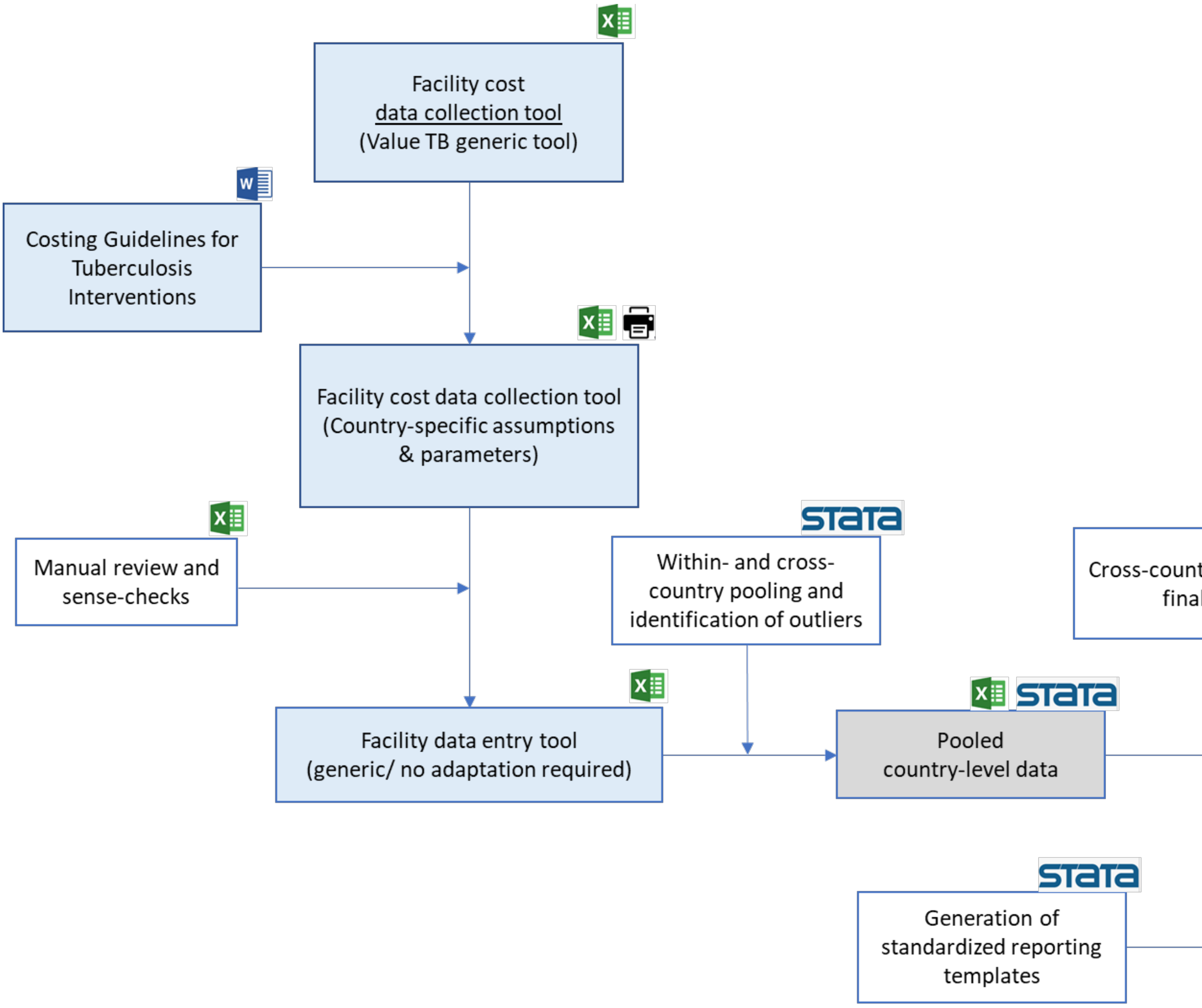

Table S1 Mean top-down unit cost estimates and coefficients of variance by service (2018 USD)

Output group and Output	Ethiopia	Georgia	India	Kenya	Philippines
**Community services**					
Community screening visit	2.60 (22%)				2.44 (0%)
Community treatment visit		3.65 (18%)	0.61 (1%)	34.80 (2%)	
Community vaccinations	2.91 (15%)			56.68 (22%)	
**Inpatient services**					
Inpatient bed-day	9.44 (11%)	27.90 (8%)	11.91 (18%)	22.97 (9%)	31.09 (9%)
**Lab tests**					
Alanine Aminotransferase	7.48 (15%)		1.42 (23%)	9.58 (0%)	
Aspartate Aminotransferase	7.68 (14%)		1.42 (23%)	8.97 (0%)	
Audiometry		4.07 (11%)		34.11 (46%)	4.41 (27%)
Blood sugar analysis	5.31 (18%)	3.09 (1%)	1.30 (11%)	6.96 (18%)	3.59 (16%)
Body fluids analysis	16.14 (32%)	4.64 (2%)		16.92 (0%)	
CD4 Count	10.79 (18%)			17.01 (19%)	
Creatinine	7.69 (16%)		1.15 (12%)	5.93 (3%)	2.33 (13%)
Drug-susceptibility tests (first-line) (liquid)		34.22 (1%)	27.87 (3%)	569.57 (0%)	
Drug-susceptibility tests (second-line) (liquid)		33.28 (1%)	27.32 (3%)		
Electrocardiogram		3.74 (11%)	1.59 (18%)	3.35 (0%)	6.16 (26%)
Electrolyte test	10.09 (22%)	4.17 (4%)			
Erythrocyte Sedimentation Rate	8.82 (16%)		1.45 (14%)	15.39 (42%)	
Full haemogram	3.86 (15%)	5.67 (6%)	1.37 (15%)	7.91 (18%)	3.86 (8%)

Haemoglobin estimation	7.10 (38%)			3.48 (15%)	
HIV confirmatory test	42.40 (37%)			11.49 (38%)	
HIV rapid test	9.93 (14%)	4.33 (8%)	1.91 (8%)	10.52 (16%)	4.65 (6%)
Light-emitting diode fluorescence microscopy (LED-FM)			1.53 (8%)	9.14 (10%)	
Lipoprotein A blood test	41.75 (3%)	76.07 (1%)	37.19 (2%)	63.95 (1%)	
Liver function test			2.50 (3%)	25.64 (16%)	4.98 (11%)
Potassium test		4.13 (6%)			2.70 (2%)
Pregnancy test			2.30 (2%)		4.21 (2%)
Protein			2.40 (2%)		1.43 (2%)
Renal function test			2.50 (3%)	22.43 (14%)	
Ziehl-Neelsen smear microscopy	8.86 (17%)	7.86 (19%)	3.95 (14%)	21.95 (30%)	6.53 (18%)
Sputum collection	6.71 (18%)	3.94 (12%)		13.96 (38%)	7.05 (8%)
Sputum culture	101.18 (21%)	21.35 (14%)	7.03 (12%)	39.49 (7%)	25.84 (4%)
Mantoux test		3.82 (2%)	2.04 (31%)		6.78 (24%)
GeneXpert MTB/RIF	29.17 (12%)	27.95 (20%)	27.94 (23%)	20.31 (15%)	29.09 (4%)
**Outpatient services**					
Outpatient cough triage	3.00 (11%)	12.34 (16%)		2.79 (15%)	1.90 (14%)
Outpatient diagnostic visit	5.06 (12%)	6.99 (7%)	2.35 (12%)	7.32 (10%)	4.13 (8%)
Outpatient monitoring visit	0.88 (2%)	7.15 (8%)			3.78 (11%)
Outpatient screening visit	4.34 (11%)	6.42 (7%)	2.87 (11%)	2.51 (9%)	4.56 (8%)
Outpatient treatment visit	5.35 (10%)	4.27 (9%)	1.93 (11%)	4.76 (8%)	3.25 (12%)
Outpatient vaccinations	5.01 (12%)		1.06 (11%)	2.63 (7%)	3.57 (10%)

**Other services**					
Contact tracing		3.98 (25%)		14.88 (24%)	
Lost to follow-up tracing	7.21 (18%)	2.70 (0%)		15.26 (15%)	1.06 (16%)
Patient support	10.21 (38%)		4.37 (12%)	50.38 (16%)	0.54 (15%)
**Radiology**					
Computerized Tomography (CT) scan		115.84 (7%)		413.28 (7%)	
Chest xray (digital)	52.62 (34%)	3.74 (11%)	4.91 (22%)	31.37 (24%)	4.23 (12%)
Chest xray (film)	8.59 (6%)	6.62 (7%)	3.16 (5%)	25.57 (30%)	6.21 (11%)
Ultrasound	14.97 (37%)	4.70 (10%)		25.68 (30%)	
Xray all other body (excluding chest xray)	6.64 (27%)	6.35 (4%)	15.52 (5%)	40.66 (25%)	

HIV human immunodeficiency virus; LED-FM light-emitting diode fluorescence microscopy; MTB mycobacterium tuberculosis; RIF rifampicin; CT computerized tomography. Services observed in only one country not shown. All costs in 2018 USD

Table S2 Mean bottom-up unit cost estimates and coefficients of variance by service (2018 USD)

Output group and Output	Ethiopia	Georgia	India	Kenya	Philippines
**Community services**					
Community screening visit	1.07 (14%)				2.02 (0%)
Community treatment visit		1.86 (4%)	0.52 (1%)	15.65 (3%)	
Community vaccinations	1.54 (23%)			52.53 (3%)	
**Inpatient services**					
Inpatient bed-day	6.87 (3%)	20.82 (14%)	7.25 (17%)	19.84 (8%)	26.83 (9%)
**Lab tests**					
Alanine Aminotransferase	4.07 (18%)		0.85 (36%)	4.38 (1%)	
Aspartate Aminotransferase	4.12 (17%)		0.85 (36%)	4.38 (1%)	
Audiometry		1.77 (7%)		4.58 (10%)	3.07 (23%)
Blood sugar analysis	3.07 (22%)	0.99 (0%)	0.80 (11%)	4.79 (14%)	2.59 (12%)
Body fluids analysis	6.90 (27%)	3.07 (3%)		5.61 (0%)	
CD4 Count	5.78 (15%)			8.00 (10%)	
Creatinine	4.20 (19%)		0.65 (15%)	5.45 (1%)	1.77 (10%)
Drug-susceptibility tests (first-line) (liquid)		19.21 (1%)	24.54 (2%)	39.94 (1%)	
Drug-susceptibility tests (second-line) (liquid)		12.18 (1%)	22.10 (2%)		
Electrocardiogram		1.93 (9%)	0.96 (22%)	2.66 (0%)	3.76 (11%)
Electrolyte test	6.44 (22%)	2.40 (3%)			
Erythrocyte Sedimentation Rate	4.94 (13%)		1.03 (13%)	6.07 (26%)	
Full haemogram	2.91 (13%)	3.94 (5%)	0.75 (15%)	5.71 (12%)	2.56 (22%)
Haemoglobin estimation	6.18 (48%)			2.89 (12%)	
HIV confirmatory test	5.29 (11%)			4.56 (37%)	
HIV rapid test	4.35 (13%)	2.77 (7%)	1.29 (8%)	5.83 (10%)	3.44 (6%)

Light-emitting diode fluorescence microscopy (LED-FM)			0.80 (12%)	9.16 (12%)	
Lipoprotein A blood test	10.44 (3%)	61.50 (0%)	18.94 (2%)	47.02 (1%)	
Liver function test			2.60 (2%)	11.74 (10%)	2.94 (7%)
Potassium test		2.42 (10%)			1.74 (2%)
Pregnancy test			1.58 (2%)		2.11 (1%)
Protein			1.81 (2%)		1.41 (1%)
Renal function test			2.05 (2%)	8.52 (21%)	
Ziehl-Neelsen smear microscopy	4.20 (13%)	4.00 (12%)	2.37 (13%)	13.28 (14%)	3.58 (13%)
Sputum collection	3.10 (16%)	1.71 (7%)		6.22 (22%)	5.63 (11%)
Sputum culture	56.01 (25%)	11.76 (17%)	11.34 (10%)	30.65 (8%)	23.37 (8%)
Mantoux test		3.44 (2%)	1.43 (38%)		3.64 (15%)
GeneXpert MTB/RIF	18.07 (2%)	14.28 (3%)	16.60 (4%)	16.69 (9%)	20.05 (2%)
**Outpatient services**					
Outpatient cough triage	1.70 (13%)	5.03 (16%)		1.79 (11%)	1.27 (14%)
Outpatient diagnostic visit	2.51 (12%)	3.11 (7%)	1.35 (12%)	4.36 (9%)	2.92 (8%)
Outpatient monitoring visit	0.80 (1%)	3.20 (9%)			2.61 (9%)
Outpatient screening visit	2.36 (12%)	2.56 (6%)	1.58 (12%)	2.24 (7%)	3.25 (7%)
Outpatient treatment visit	2.78 (11%)	2.03 (8%)	1.09 (11%)	4.06 (6%)	2.29 (10%)
Outpatient vaccinations	2.03 (11%)		0.67 (10%)	2.13 (6%)	2.24 (10%)
**Other services**					
Contact tracing		2.67 (27%)		13.15 (29%)	
Lost to follow-up tracing	3.46 (20%)	1.22 (0%)		13.59 (17%)	0.58 (11%)
Patient support	7.68 (41%)		2.11 (16%)	42.34 (14%)	0.39 (12%)
**Radiology**					

Computerized Tomography (CT) scan		22.47 (3%)		17.61 (1%)	
Chest xray (digital)	9.86 (17%)	2.01 (24%)	2.78 (19%)	23.02 (28%)	2.94 (11%)
Chest xray (film)	5.51 (6%)	4.34 (5%)	1.89 (6%)	14.21 (22%)	3.36 (5%)
Ultrasound	8.13 (27%)	2.71 (14%)		9.10 (16%)	
Xray all other body (excluding chest xray)	3.07 (32%)	5.15 (4%)	7.62 (6%)	25.00 (35%)	

HIV human immunodeficiency virus; LED-FM light-emitting diode fluorescence microscopy; MTB mycobacterium tuberculosis; RIF rifampicin; CT computerized tomography. Services observed in only one country not shown. All costs in 2018 USD
